# Evaluation of diagnostic accuracy of CBCT and intraoral radiography for proximal caries detection in the presence of different dental restoration materials

**DOI:** 10.1186/s12903-023-02954-8

**Published:** 2023-06-23

**Authors:** Farzaneh Mosavat, Elham Ahmadi, Saba Amirfarhangi, Niyousha Rafeie

**Affiliations:** 1 Oral and Maxillofacial Radiology Department, School of Dentistry, Tehran University of Medical Science, Tehran, Iran; 2 Department of Operative Dentistry, Dental Research Center, Dentistry Research Institute, School of Dentistry, Tehran University of Medical Sciences, Tehran, Iran; 3 Department of Prosthodontics, Dental Faculty, Qazvin University of Medical Sciences, Qazvin, Iran; 4 Dental Research center, Dentistry research institute, School of Dentistry, Tehran University of Medical Sciences, Tehran, Iran

**Keywords:** Cone-Beam Computed Tomography, Artifact, Digital radiography, Dental Porcelain

## Abstract

**Purpose:**

This study aimed to assess the diagnostic accuracy of cone-beam computed tomography (CBCT) and digital intraoral radiography for the detection of proximal caries adjacent to amalgam, e.max porcelain, and metal-ceramic restorations (MCRs).

**Materials and methods:**

Parallel intraoral radiographs were obtained from 40 posterior teeth using PSP sensors. To obtain CBCT scans, the teeth were first radiographed alone, and were then positioned next to a tooth with an amalgam restoration, MCR, and e.max porcelain crown, and radiographed again. Two blinded observers scored radiographs using a four-point scale (0: absence of proximal caries, 1: enamel caries, 2: carious lesion extending to the outer half of dentin, 3: carious lesion extending to the inner half of dentin). Tooth sections were made, and the grade of caries was determined under a light microscope at x12 magnification. The sensitivity, specificity, and accuracy of CBCT and intraoral radiographs were then calculated.

**Results:**

Artifact-free CBCT scans and intraoral radiographs had the highest diagnostic accuracy (0.826 and 0.657, respectively) while CBCT images of the teeth next to the amalgam restorations (0.526) had the lowest accuracy. The diagnostic accuracy of CBCT images of the teeth next to the porcelain crowns and MCRs was 0.613 and 0.601, respectively.

**Conclusion:**

Artifact-free CBCT images had higher diagnostic accuracy than intraoral radiography for the detection of all grades of proximal caries. The diagnostic accuracy of CBCT images of teeth adjacent to amalgam, porcelain, and MCRs was lower compared to intraoral radiographs and artifact-free CBCT images.

**Supplementary Information:**

The online version contains supplementary material available at 10.1186/s12903-023-02954-8.

## Introduction

Dental caries is an infectious microbial disease of the tooth structure causing dissolution and demineralization of the mineral parts of the tooth and it is the most common chronic oral disease. Dental clinicians often detect proximal caries by visual inspection and intraoral radiography [1]. Among intraoral radiographic techniques, bitewing is the most commonly used radiograph for the detection of proximal caries in posterior regions [2]. However, according to the literature, bitewing radiography can detect roughly 60-85% of proximal caries [3, 4]. Moreover, it is believed that caries lesions should undergo at least 40% mineral loss to be radiographically visible [5], and thus non-cavitated lesions with less than 40% mineral loss might not be detected on intraoral radiographs. As a result, these lesions progress to larger lesions over time, causing more extensive tooth loss [6]. The early and precise detection of proximal carious lesions is of significant concern since it enables clinicians to provide immediate operative procedures, preventing further tooth loss [7].

In addition, intraoral radiography is two-dimensional which limits the detection of proximal caries in the three-dimensional tooth structure.

In recent decades, many researchers have proposed Cone Beam Computed Tomography (CBCT) as a valuable additional aid in the detection of proximal caries. CBCT images are free of distortion, superimposition, and magnification [8]. Easy use, 3D image reconstruction, and providing different sections in multiple planes are some of the other advantages of CBCT images. Thus, by using CBCT, beneficial information can be obtained qualitatively and quantitatively through a slight increase in radiation dose [9].

However, the diagnostic accuracy of CBCT images in the detection of proximal caries is controversial. Several studies have reported better sensitivity, specificity, and diagnostic accuracy of CBCT images in comparison with intraoral radiographs when there is no adjacent metal restoration [10–12]. On the other hand, some studies have reported similar or less diagnostic accuracy of CBCT images compared to conventional and digital intraoral modalities regarding proximal caries detection [5, 12–15].

Dentists use various restorative materials with variable levels of radiopacity [8]. These materials can be seen as artifacts- bright streaks and dark bands-on CBCT images due to the beam hardening phenomenon. This phenomenon occurs by the interaction between polyenergetic X-rays and high-density restorative materials [16, 17]. These artifacts might interfere with the interpretation and detection of carious lesions by reducing contrast and concealing structures [8].

Due to the limited number of studies investigating the diagnostic accuracy of CBCT images regarding proximal caries in the presence of different restorative materials, the constant influx of newly introduced dental materials with different X-ray attenuating characteristics and also the increased application of CBCT images in dental practice, this study aimed to evaluate the diagnostic accuracy of cone-beam computed tomography (CBCT) and digital intraoral radiography for the detection of proximal caries adjacent to amalgam, e.max porcelain, and metal-ceramic restorations (MCRs).

## Materials and methods

The present experimental *in-vitro* study (approved by the ethics committee of the Tehran University of Medical Sciences, School of Dentistry, code of ethics: 6352) was performed on forty cavitated and non-cavitated permanent posterior teeth that had been extracted for orthodontic or periodontal purposes.

Given that 50% of the teeth are carious in a certain population, and radiography can correctly detect a minimum of 65% of the carious lesions according to the criteria by the World Health Organization, and assuming alpha = 0.05, beta = 0.2, and study power of 80%, a minimum of 75 tooth surfaces would be required for this study. Since each tooth has two proximal surfaces, a total of 80 surfaces were evaluated in this study.

The teeth were selected using convenience sampling irrespective of the age and gender of the patients and had no cracks, restorations, or fractures. Soft tissue residues were removed, and the teeth were stored in distilled water and refrigerated at -4 °C until use. For the purpose of disinfection, the teeth were immersed in chloramine T solution (Merck, Germany) for one week and were then coded randomly. Two teeth were first mounted in tooth sockets in a dry mandible, and the presence of interdental contact was ensured by using dental floss. To simulate the effects of soft tissue, a wax block (10 × 5 cm) was used in front of the teeth.

**Fig. 1:**
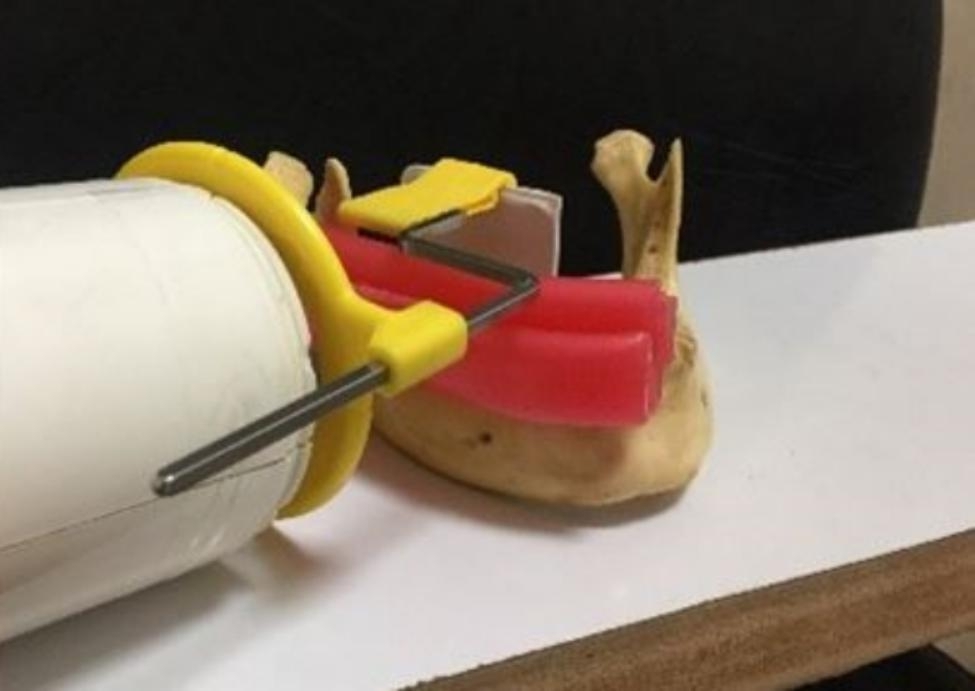
The mandible and paralleling film holder were fixed on a wooden board to standardize the radiation angle, tooth position, and position of the image receptor

The digital intraoral radiographs were obtained by the parallel technique using a dental X-ray unit (Owandy RX, France) with the exposure settings of 60 kVp and 0.32 s exposure time and a PSP scanner (Digora Optime, Soredex, Finland). Each radiograph was coded according to the coding of the respective tooth. During the procedure, the radiation angle, tooth position, and position of the image receptor (PSP sensor) were fixed using a wooden board (20 × 20 cm) on which, the body of the mandible and the extension cone paralleling film holder were fixed (Fig. 1). The dry mandible was placed on the wooden board such that the occlusal plane of the mounted teeth was parallel to the horizon and the radiation angle was adjusted perpendicular to the longitudinal axis of the tooth.

**Fig. 2:**
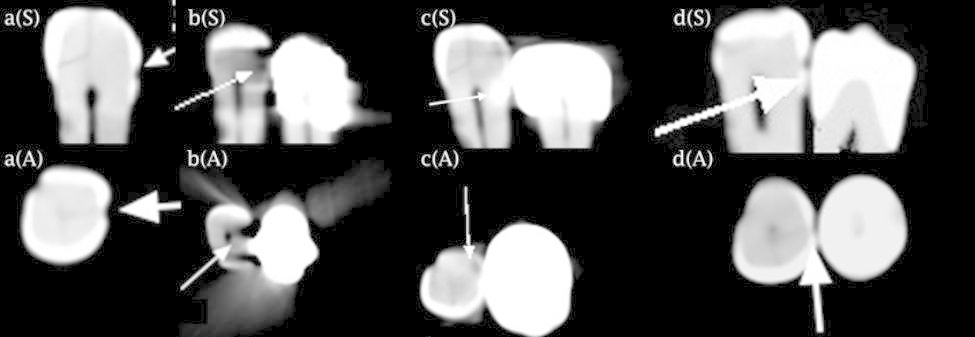
Sagittal (S-upper row) and axial (A-lower row) scans of (a) a tooth with enamel caries pointed by the arrow, the same tooth next to the (b) amalgam restoration, (c) metal-ceramic restoration, and (d) porcelain crown. In images b(S) and b(A), a metal artifact (arrow) caused by amalgam is seen as a radiolucent area resembling dentin caries. In image c(S), the artifact (arrow) caused by metal-ceramic restoration is seen as an area with high attenuation, covering the carious lesion. In image c(A), streak-artifact (arrow) caused by metal-ceramic restoration is seen as a lucent area resembling dentin caries

**Fig. 3:**
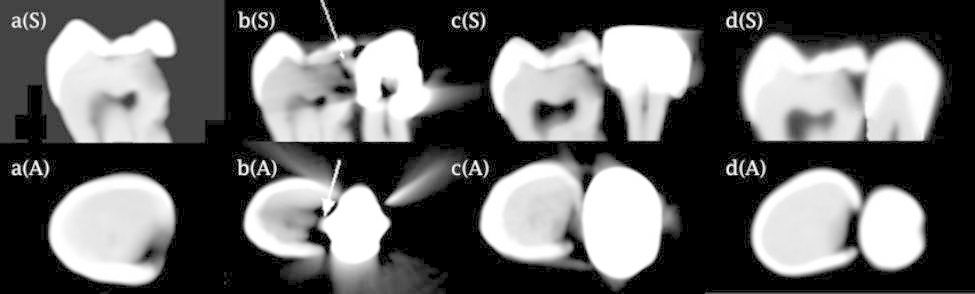
Sagittal (S-upper row) and axial (A-lower row) scans of (a) a tooth with dentin caries pointed by the arrow, the same tooth next to the (b) amalgam restoration, (c) metal-ceramic restoration, and (d) porcelain crown. In images b(S) and b(A), the cupping artifact (arrow) caused by amalgam is seen as a radiopaque area which can interfere with caries detection

To obtain CBCT scans, the dry mandible along with two layers of wax (for soft tissue simulation) was placed on a wooden board at the center of the field of view of the scanner. The occlusal plane of the mounted teeth was parallel to the horizon (as for intraoral radiography). Each tooth was first radiographed alone, and then was placed next to a tooth with an amalgam restoration (high copper admixed; Cinalux, Iran), a tooth with MCR (Ivoclar Vivadent, Schaan, Lichtenstein), and a tooth with lithium disilicate porcelain crown (e.max CAD; Ivoclar Vivadent, Schaan, Lichtenstein) respectively. Each time, one radiograph was taken. This process was performed separately for the mesial and distal surfaces of the teeth. Figures 2 and 3 show CBCT scans of the teeth alone, placed next to a tooth with an amalgam restoration, a MCR, and a lithium disilicate porcelain crown.

The CBCT scans were obtained using an Alphared-3030 CBCT scanner (Asahi Roengten. Ind. Co., Kyoto, Japan) with the exposure settings of 10 × 10 cm field of view, 17 s time, 4 mA amperage, 80 kV voltage, and 0.2 mm voxel size. The CBCT scans were then reconstructed using the system software. Data were transferred to Romexis software (Planmeca Oy, Helsinki, Finland). Eventually, 40 digital intraoral radiographs, 40 CBCT scans without artifacts, and 240 CBCT scans with artifacts were obtained.

The intraoral radiographs were coded from 1 to 40 while the CBCT scans were randomly coded from 1 to 280. Both mesial and distal surfaces of the teeth were evaluated on intraoral radiographs and artifact-free CBCT scans. However, only the proximal surface of the tooth in contact with the adjacent tooth was evaluated on CBCT scans with artifacts. Totally, 80 tooth surfaces were evaluated on intraoral radiographs, and 320 tooth surfaces were evaluated on CBCT scans for the detection of caries. The obtained digital intraoral and CBCT images were observed by two general dentists blinded to the coding of the radiographs in a poorly-lit room using Soredex and Romexis software programs, and the results were recorded in a checklist. The observers were allowed to change the contrast and brightness of the images and had no time limitation for the evaluation of radiographs. They could also zoom the images, use magnification, and evaluate different planes of 3D images.

**Fig. 4:**
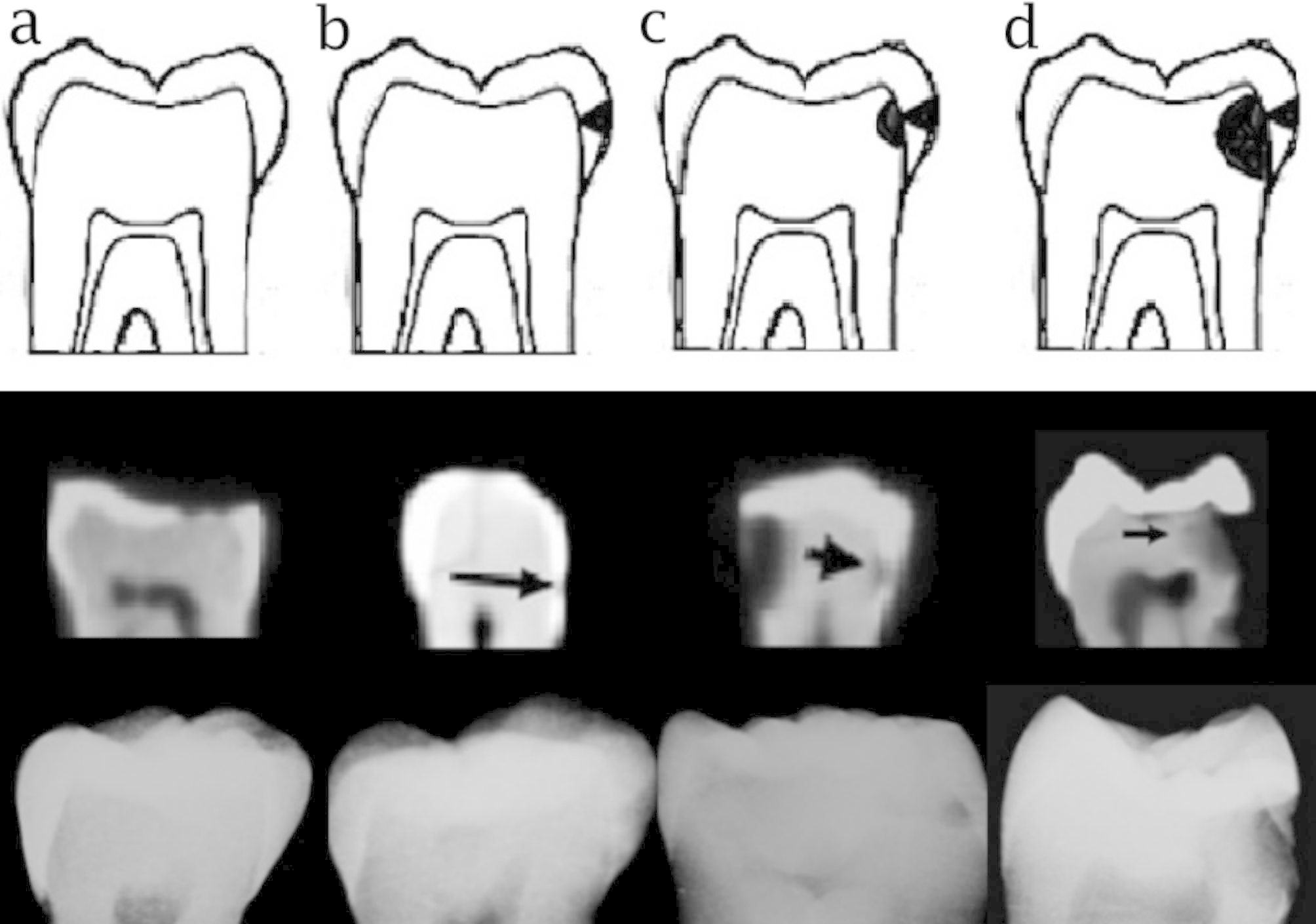
Schematic (upper row), CBCT (middle row), and intra-oral X-ray images (lower row) of the teeth; (a) absence of proximal caries (score 0), (b) enamel caries (score 1), (c) carious lesion extending to the outer half of dentin (score 2), and (d) carious lesion extending to the inner half of dentin (score 3)

The presence/absence of proximal caries was scored using a four-point scale as follows: 0: absence of proximal caries, 1: enamel caries, 2: carious lesion extending to the outer half of dentin, and 3: carious lesion extending to the inner half of dentin (Fig. 4).

**Fig. 5:**
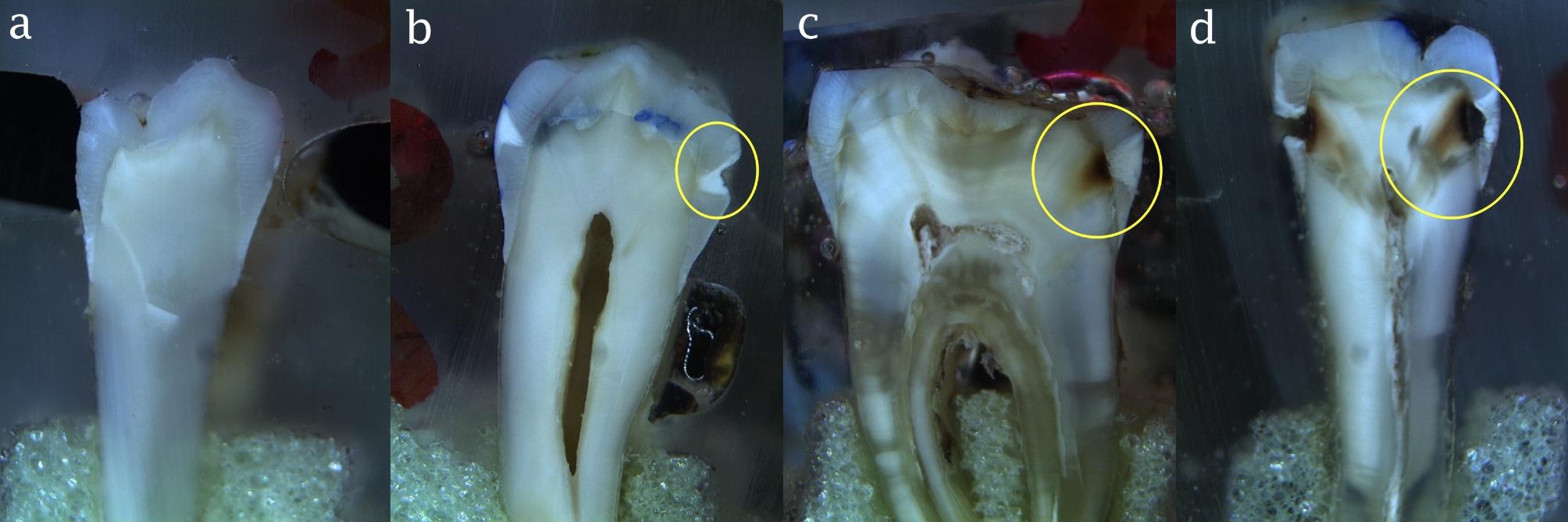
Images of teeth sections under a light microscope with ×12 magnification; (a) absence of proximal caries (score 0), (b) enamel caries (score 1), (c) carious lesion extending to the outer half of dentin (score 2), and (d) carious lesion extending to the inner half of dentin (score 3). The yellow circle shows the carious lesion

Microscopic evaluation of the teeth was then performed to serve as the gold standard (actual presence/absence of caries). For this purpose, the teeth were embedded in resin and sectioned mesiodistally by a 500-µm diamond saw (T210; Mecatome, Presi, France) to obtain 500 μm slices. The sections were inspected by one observer under a light microscope (SZX16, Olympus, Tokyo, Japan) at ×12 magnification (Fig. 5). The presence of caries was confirmed in case of the presence of decalcified white regions or brown regions on the proximal side of the pulp chamber. Accordingly, tooth sections were assigned to four groups of sound, enamel caries, carious lesion in the outer half of dentin, and carious lesion in the inner half of dentin.

Data regarding the observers opinions on the presence/absence of proximal caries on intraoral radiographs, CBCT scans of the teeth alone, and CBCT scans of the teeth along with adjacent restorations (amalgam restorations, MCRs, and porcelain crowns) as well as microscopic analysis results were collected. Data were analyzed using SPSS version 25. Also, the sensitivity and specificity values were calculated for each group of images separately for each observer. The inter-observer agreement for each group of images was also calculated using the kappa coefficient. The acceptable error rate was 5% in this study.

## Results

According to histological findings (gold standard), the frequency of sound surfaces (score 0), enamel caries (score 1), carious lesions in the outer half of dentin (score 2), and carious lesions in the inner half of dentin (score 3) was 28, 9, 25 and 18, respectively.

Table 1 shows the inter-observer agreement for different image groups.

**Table 1:** Inter-observer agreement (kappa coefficient) for detection of dental caries on intraoral radiographs and CBCT scans

	intraoral radiography	CBCT	CBCT next to amalgam restoration	CBCT next to MCR	CBCT next to porcelain crown
Kappa coefficient	**0.827**	**0.929**	**0.683**	**0.701**	**0.924**

According to the sensitivity and specificity evaluations, artifact-free CBCT scans and intraoral radiographs had the highest diagnostic accuracy (0.826 and 0.657, respectively) while CBCT images of the teeth next to amalgam restorations had the lowest accuracy (0.526). The diagnostic accuracy of CBCT images of the teeth next to porcelain crowns and MCRs was 0.613 and 0.601 respectively (Tables 2 and 3).

**Table 2:** Sensitivity and specificity of intraoral radiography and CBCT scans (with and without artifacts) for detection of proximal caries by the two observers

Diagnostic parameters	Observers	Specificity	Mean	Sensitivity (enamel caries)	Mean	Sensitivity (caries in the outer half of dentin)	Mean	Sensitivity (caries in the inner half of dentin)	Mean	General sensitivity	Mean
intraoral	First	**0.929**	**0.911**	**0.333**	**0.389**	**0.72**	**0.72**	**0.278**	**0.306**	**0.769**	**0.789**
	Second	**0.893**		**0.444**		**0.72**		**0.333**		**0.808**	
artifact-free CBCT	First	**0.929**	**0.947**	**0.889**	**0.833**	**0.84**	**0.84**	**0.667**	**0.612**	**0.942**	**0.942**
	Second	**0.964**		**0.778**		**0.84**		**0.556**		**0.942**	
CBCT next to amalgam	First	**0.214**	**0.268**	**0.556**	**0.50**	**0.84**	**0.88**	**0.444**	**0.444**	**0.962**	**0.952**
	Second	**0.221**		**0.444**		**0.92**		**0/444**		**0/942**	
CBCT next to MCR	First	**0.75**	**0.697**	**0.333**	**0.333**	**0.72**	**0.72**	**0.444**	**0.417**	**0.808**	**0.817**
	Second	**0.643**		**0.333**		**0.72**		**0.389**		**0.827**	
CBCT next to porcelain crown	First	**0.786**	**0.786**	**0.444**	**0.444**	**0.64**	**0.64**	**0.389**	**0.389**	**0.788**	**0.788**
	Second	**0.786**		**0.444**		**0.64**		**0.389**		**0.788**	

**Table 3:** Accuracy of detection of proximal caries on intraoral radiographs and CBCT scans with and without artifacts by the observers

observer	intraoral	Mean	Artifact-free CBCT	Mean	CBCT next to amalgam restoration	Mean	CBCT next to MCR	Mean	CBCT next to porcelain crown	Mean
**First**	0.651	0.657	0.838	0.826	0.501	0.526	0.626	0.601	0.613	0.613
**Second**	0.663		0.814		0.551		0.526		0.613	

## Discussion

This in-vitro study compared the accuracy of artifact-free CBCT images, and CBCT images with artifacts (caused by the adjacent amalgam, e.max porcelain, and MCRs) with the accuracy of PSP digital intraoral radiography for the detection of proximal caries. The results showed that artifact-free CBCT images had the highest diagnostic specificity followed by PSP digital intraoral radiography. The diagnostic specificity of CBCT images of the teeth next to the amalgam restorations significantly decreased (0.268). This value was even lower than that of CBCT images of the teeth next to MCRs (0.697) and porcelain crowns (0.786). This finding indicates that although CBCT images have maximum accuracy for detection of sound teeth, the presence of adjacent high-density restorative materials such as amalgam would cause metal artifacts and errors in the correct detection of sound surfaces, increasing the false positive results.

The specificity of CBCT and PSP digital radiography for the detection of dental caries was 0.97 and 0.90 respectively. In studies conducted by Gaalaas et al. and Haither et al., these values were 0.89 and 0.91 respectively which were close to the current findings [18, 19]. However, in studies by Kalathingal et al. [20] and Belem et al. [21], the specificity of CBCT images was 0.82 and 0.867 respectively and the specificity of PSP digital images was reported to be 0.879 in the Belem et al. ^21^study. It should be noted that the sample size was 20 in the Belem et al. study which was smaller than our sample size. Moreover, the rotation of the CBCT scanner was 180° in their study while this value was 360° in our study. By decreasing the scanning arc to 180°, the signal/noise ratio decreases, which results in the subsequent reduction of diagnostic accuracy of images.

Wenzel et al. reported that the specificity of CBCT and PSP digital radiography ranged from 98 to 100%, which was higher than the specificity range in our study. Visual inspection for the detection of caries without histological examination, which served as the gold standard in their study, may explain the differences in the results [10].

In this study, the artifact-free CBCT images had maximum sensitivity for the detection of caries (0.942) followed by the CBCT images of the teeth adjacent to amalgam restorations (0.952). The minimum values belonged to intraoral radiography and CBCT images of the teeth adjacent to porcelain crowns (0.788). The diagnostic sensitivity of CBCT and PSP radiography for the detection of caries in the study by Belem et al. [21] was 0.880 and 0.654 respectively which were close to the values obtained in the present study. In the studies conducted by Kalathingal et al. [20], Gaalaas et al. [18], Wenzel et al. [10], and Haither et al. [19], the diagnostic sensitivity of CBCT images for the detection of caries was 0.70, 0.62, 0.40, and 0.21 respectively while these values were 0.57, 0.17, 0.17 for PSP images respectively. Although the values obtained in the abovementioned studies were lower than the values in our study, CBCT still showed higher sensitivity than PSP radiography for caries detection [10, 20].

A significant difference between the sensitivity of CBCT and intraoral radiography indicates that many incipient carious lesions remain undetected on intraoral radiographs due to inappropriate image angulation or overlap of contact areas. Moreover, dental caries is visualized on an intraoral radiograph only when 30–60% demineralization of the tooth structure has occurred [4]. However, artifact-free CBCT scans can reveal incipient caries. It should be noted that the presence of artifacts on CBCT images significantly increases the possibility of false positive results. As shown in Table 2, although the sensitivity of CBCT for caries detection next to amalgam restorations is very high, its low specificity is responsible for the false positive results, which would lead to unnecessary restorative treatments.

Kalathingal et al. [20] reported that the observers had superior performance in the detection of the depth of proximal carious lesions on CBCT scans. In their study, CBCT showed higher sensitivity than film-based radiography in the overall detection of caries and detection of the depth of lesions. In our study, the diagnostic sensitivity of artifact-free CBCT scans was higher for detection of grade 1, 2 and 3 caries by both observers than other images. The artifact-free CBCT images had maximum sensitivity for the detection of enamel caries (0.833). However, the sensitivity of other images was not acceptable for the detection of enamel caries because it was maximally 50%.

For detection of caries in the outer half of dentin, CBCT images of the teeth next to amalgam restorations had maximum sensitivity (0.88) followed by artifact-free CBCT images (0.84); however, the difference with other images was not significant. This finding is justifiable considering the fact that the presence of a radiolucency at the dentin-enamel junction is a characteristic landmark for the detection of carious lesions in the outer half of dentin. However, the high sensitivity of images adjacent to an amalgam restoration can be due to the presence of lucent artifacts caused by the high-density material and also the match band effect (considering the low specificity), rather than the higher accuracy of caries detection.

For caries in the inner half of dentin, the minimum sensitivity value belonged to intraoral radiography (0.301) while the maximum value belonged to artifact-free CBCT (0.612). It should be noted that intraoral radiography can detect caries with at least 30–60% demineralization; thus, it always underestimates the extent of carious lesions. The possibility of detecting caries with a lower rate of demineralization is higher on CBCT images, which can explain the lower diagnostic sensitivity of intraoral radiography compared to that of CBCT images for caries detection in the inner half of dentin.

In this study, CBCT and intraoral radiography had maximum diagnostic accuracy (0.826 and 0.657 respectively) while CBCT images of the teeth adjacent to amalgam restorations had minimum accuracy (0.526). In the studies by Cheng et al. [11], Zhang et al. [12], and Wenzel et al. [10], the diagnostic accuracy of CBCT images was found to be slightly higher than that of PSP digital radiography which was in agreement with the current results. In studies by Senel et al. [5] and Kayipmaz et al. [13], PSP digital radiography and CBCT had similar diagnostic accuracy for the detection of proximal caries, which was different from our findings. Lower voltage (kVp) of CBCT scanners can decrease their diagnostic accuracy (70 kVp). Also, thicker histological Sect. (0.4 mm), compared with the present study, can be responsible for lower diagnostic accuracy. Lower diagnostic accuracy for caries detection in teeth adjacent to amalgam restorations or MCRs can be related to the formation of artifacts. Metal artifacts-as the result of beam hardening-can cause dark bands and streaks on radiographs and lead to false positive and false negative results [22].

It should be noted that the majority of relevant previous studies [5, 10–13, 18, 21] have compared the diagnostic accuracy of CBCT and intraoral radiography for caries detection, and studies on the effect of adjacent restoration artifacts on the diagnostic accuracy of caries detection in different parts of the tooth are limited. Kulczyk et al. [23] evaluated the effect of adjacent amalgam restorations on the diagnostic accuracy of CBCT for caries detection and reported a diagnostic specificity of 0.52, which was higher than the value in our study (0.26). Cebe et al. [24] evaluated the effect of artifacts of restorative materials on the detection of proximal caries on CBCT scans and reported a specificity of 0.05 and sensitivity of 0.96 for the detection of caries at the contact area of the tooth and amalgam restoration. Diagnostic sensitivity and specificity for caries detection in surfaces adjacent to all-ceramic crowns were 0.97 and 0.21 respectively in their study. Their reported values were different from the findings of the current study, which may be due to the different materials of prosthetic crowns (e. max in our study and zirconia in their study). Also, the voltage of CBCT scanners can affect the number and intensity of formed artifacts. Moreover, the degree of rotation of the scanner, and the type of software program can also affect the rate and intensity of artifacts [25, 26].

The diagnostic specificity and sensitivity of artifact-free CBCT images for the detection of proximal caries were higher than those of intraoral radiography. Imaging of the teeth next to porcelain crowns and MCRs had lower diagnostic parameters for proximal caries detection. However, the difference between MCR and porcelain was not significant in this respect. Thus, it may be concluded that the thinness of the metal core in MCRs and the high atomic number of porcelain are probably responsible for equal amounts of artifacts seen in tooth images next to MCR and porcelain crowns.

In this study, only artifact-free CBCT images showed high diagnostic specificity. The diagnostic specificity of digital intraoral radiography was inferior to artifact-free CBCT images but was still acceptable. The diagnostic specificity of CBCT images decreased in teeth adjacent to porcelain crowns and MCRs and was minimal adjacent to the amalgam restorations. According to the literature, the detection of sound teeth next to amalgam restorations is much more difficult than the detection of sound teeth adjacent to a ceramic crown or MCR on CBCT images. Since the beam hardening effect is highly correlated with the density and the atomic number of materials, the presence of amalgam causes greater artifacts and mimics carious lesions while ceramic crowns and MCRs cause smaller artifacts; thus, the diagnostic results adjacent to MCRs or ceramic crowns are more accurate.

Future studies are required to investigate the effects of different exposure parameters of CBCT such as voltage, amperage, and field of view on the accuracy of caries detection. Also, different CBCT scanners should be compared regarding their diagnostic efficacy for caries detection in presence of metal restorations.

In conclusion, artifact-free CBCT scans had maximum diagnostic accuracy for the detection of proximal caries followed by intraoral radiography, and CBCT scans of the teeth adjacent to porcelain crowns, MCRs, and amalgam restorations. Thus, interpretation of CBCT images of the teeth adjacent to amalgam restorations should be done with caution to prevent false positive diagnosis. Regarding the detection of enamel caries, artifact-free CBCT images showed maximum diagnostic sensitivity while the sensitivity of other imaging modalities, even intraoral radiography, was not acceptable for this purpose. For the detection of caries in the outer half of dentin, intraoral radiography had maximum sensitivity followed by artifact-free CBCT images. In addition, artifact-free CBCT was more accurate for the detection of the depth of caries.

It should be noted that using CBCT to detect proximal caries is not justifiable by ALARA (as low as reasonably achievable radiation) and we cannot recommend CBCT as a method for caries detection. However, CBCT might be helpful to detect caries in patients who have already taken CBCT for implant placement or other reasons since artifact-free CBCT images had higher diagnostic accuracy than intraoral x-ray radiography for detection of all grades of proximal caries as shown in the present study.

## Electronic supplementary material

Below is the link to the electronic supplementary material.

Supplementary Material 1

## Data Availability

All data generated or analysed during this study are included in this published article and supplementary file 1.
